# Enhancing the Stability and Anticancer Activity of *Escherichia coli* Asparaginase Through Nanoparticle Immobilization: A Biotechnological Perspective on Nano Chitosan

**DOI:** 10.3390/polym16233260

**Published:** 2024-11-23

**Authors:** Fahad Alharthi, Hussam A. Althagafi, Ibrahim Jafri, Atif Abdulwahab A. Oyouni, Mohammed M. Althaqafi, Nawal E. Al-Hazmi, Layla Yousif Abdullah Al Hijab, Deyala M. Naguib

**Affiliations:** 1 Department of Biology, College of Science, Taif University, P.O. Box 11099, Taif 21944, Saudi Arabia; f.alharthi@tu.edu.sa; 2 Department of Biology, Faculty of Science, Al-Baha University, Al-Baha 65525, Saudi Arabia; halthaqafi@bu.edu.sa (H.A.A.); lalhejab@bu.edu.sa (L.Y.A.A.H.); 3 Department of Biotechnology, College of Science, Taif University, P.O. Box 11099, Taif 21944, Saudi Arabia; i.jafri@tu.edu.sa (I.J.); mm.mohammad@tu.edu.sa (M.M.A.); 4 Department of Biology, Faculty of Science, University of Tabuk, Tabuk 71491, Saudi Arabia; a.oyouni@ut.edu.sa; 5 Biodiversity Genomics Unit, Faculty of Science, University of Tabuk, Tabuk 71491, Saudi Arabia; 6 Department of Chemistry, Division of Biology (Microbiology), University College of Qunfudah, Umm Al-Qura University, Qunfudah 21961, Saudi Arabia; nehazmi@uqu.edu.sa; 7 Botany and Microbiology Department, Faculty of Science, Zagazig University, Zagazig 44511, Egypt

**Keywords:** catalytic efficiency, nano-chitosan, Michaelis constants (K_m_), maximum velocity (V_max_), secondary structure, specificity constant, nuclear intensity, mitochondrial membrane

## Abstract

There is a shortage in the experimental research directly comparing the effectiveness of different nanoparticles in boosting asparaginase (ASNase) activity. This study assessed the impact of various nanoparticles on enhancing ASNase activity, stability, and anticancer effects through immobilization. *Escherichia coli* ASNase was immobilized on different nanoparticles, and its efficiency was measured. The research included analyzing the enzyme’s secondary structure, stability, activity at different temperatures, kinetic parameters, shelf life, and activity in blood serum. The anticancer efficacy was determined by measuring the IC_50_. The study also investigated the anticancer mechanisms by examining the enzyme’s toxicity on cancer cells, focusing on apoptosis indicators like nuclear intensity, membrane permeability, mitochondrial membrane permeability, and cytochrome c release. Among the tested nanoparticles, nano chitosan yielded the best improvements. ASNase immobilized on nano chitosan reached 90% immobilization efficiency fastest among the studied nanoparticles, achieving this within 72 h, whereas other nanoparticles took 120 h. Immobilization modified ASNase’s secondary structure by increasing alpha helices and reducing random coils, with nanochitosan and magnetic iron oxide showing the most pronounced effects. Immobilized ASNase exhibited enhanced activity, stability across temperature (widest with nanochitosan, 25–65 °C), and a broader optimal pH range compared to the free enzyme, with a K_m_ of 1.227 mM and a V_max_ of 454.54 U/mg protein. Notably, the nano-chitosan-immobilized ASNase retained over 85% of its activity after 9 months of storage and maintained high activity in blood serum. This improved stability and activity translated into the highest anticancer activity (Lowest IC_50_) and was more effective than doxorubicin in disrupting cancer cell structures.

## 1. Introduction

The ongoing challenges in cancer therapy, such as the toxicity and side effects of chemotherapy, drug resistance, and high costs, highlight the necessity for new and innovative anticancer agents [1,2]. By advancing precision medicine, immunotherapy, nanotechnology, enzyme therapy, and other cutting-edge approaches, the goal is to develop treatments that are not only more effective but also safer, with fewer side effects, improving the quality of life for cancer patients worldwide [3,4,5,6]. Enzymes have emerged as a promising tool in cancer therapy due to their ability to catalyze specific biochemical reactions with high efficiency and specificity. They can be used directly or as part of more complex therapeutic strategies. There are various enzymes that are used as anticancer agents, such as asparginase (ASNase, EC 3.5.1.1), arginine deiminase, methionase, lysine oxidase, phenylalanine ammonia lyase, and glutaminase [7,8,9]. These amino acid-degrading enzymes represent a unique and promising approach in cancer therapy, targeting the metabolic dependencies of cancer cells. Many cancer cells are unable to synthesize certain amino acids due to genetic or metabolic defects. The enzymatic depletion of these amino acids prevents the synthesis of proteins necessary for cancer cell growth and division, leading to cell death. Amino acid starvation can induce cell cycle arrest at various checkpoints (e.g., G1 phase), preventing cancer cells from proliferating. The deprivation of essential amino acids triggers apoptotic pathways in cancer cells. This can involve the activation of stress response pathways and pro-apoptotic factors. Amino acid depletion can affect the tumor microenvironment, influencing immune cell infiltration and tumor angiogenesis [10,11,12,13].

Recent studies try to increase the effectiveness of these amino acid degrading enzymes through immobilization on different materials. Immobilization techniques enhance enzyme stability, reusability, and overall efficiency. Nano materials have gained significant attention for enzyme immobilization due to their unique properties, such as a high surface area, tunable surface chemistry, and an enhanced mass transfer [14,15,16]. Many nano materials are used for immobilization of the therapeutic enzymes. Immobilizing proteolytic enzymes on mesoporous silica nanoparticles enhances their tumor penetration [17]. Polyamidoamine dendrimers functionalized with ZnO-chitosan nanoparticles are used for immobilizing L-ASNase to enhance its anticancer efficacy [18].

ASNase was the first microbial enzyme approved as a monotherapy for treating pediatric acute lymphoblastic leukemia. It catalyzes the conversion of L-asparagine to aspartate, releasing ammonia [19,20,21]. L-asparagine is essential for protein synthesis, the bioregulation of certain oncogenic transcription factors, and the proper functioning of the immune and nervous systems. Normal cells produce L-asparagine using asparagine synthetase, but some malignant cells, such as leukemic cells, have an inadequate expression of this enzyme and depend on external sources of L-asparagine. Therefore, depleting L-asparagine disrupts cellular signaling pathways and inhibits the expression of various oncogenic transcription factors, leading to the regression of asparagine-auxotrophic tumors [22,23,24,25]. ASNase has been isolated from various microbes, including bacteria, filamentous fungi, yeast, and algae. Various studies have tried to improve ASNase as an anticancer agent through its immobilization on different nanoparticles [26]. However, there are no experimental studies comparing the effectiveness of these nanoparticles in improving the ASNase activity. Therefore, we tried, in this study, to evaluate the differences between different kinds of nanoparticles in improving ASNase activity and to examine its anticancer activity through enzyme immobilization.

## 2. Materials and Methods

### 2.1. Immobilization of ASNase on Various Nanoparticles

#### 2.1.1. Source of ASNase

The ASNase utilized in this research was obtained from Sigma-Aldrich, Munich, Germany, specifically, ASNase from *Escherichia coli*, lyophilized powder, 100–300 U/mg protein (A3809).

#### 2.1.2. Source of Nanoparticles

The nanoparticles used were acquired from Nanochemazone, including chitosan nanoparticles (NCZ-MN-116/20), spherical gold nanoparticles (NCZ-ST-192/22), silver nanoparticles (NCZ-ST-271/22), magnetic iron(III) oxide nanoparticles (NCZ-ST-128/23), and mesoporous silica nanoparticles (NCZ-NP-620/24) (Supplementary data Tables S1–S5).

#### 2.1.3. Immobilization Procedure

The immobilization of ASNase onto nanoparticles followed the end-over-end method described by Naguib and Badawy [27]. Known weights of the studied nanoparticles (to form stable suspension) were incubated with an ASNase solution (100 mg/mL), a high-enzyme concentration that, in immobilization, enhances enzyme loading and immobilization efficiency, leading to greater catalytic activity and stability. It also reduces enzyme leakage and helps overcome mass transfer limitations, ensuring better overall performance in the immobilized system) at pH 7 and room temperature (for enzyme stability) for varying incubation periods. Post-incubation, the mixture was centrifuged at 4000× *g* for 10 min. The resulting precipitate was dried (nano ASNase) and the supernatant was retained to assess the immobilization yield. The efficiency of immobilization was calculated using the relationship proposed by Huang et al. [28].

(1) $$Immobilization\;efficiency\;\left(\%\right)=\frac{Initial\;content\;of\;protein-Final\;content\;of\;protein}{Initial\;content\;of\;protein}\times 100$$

### 2.2. Physiochemical Characterization of ASNase

#### 2.2.1. Enzyme Assay

ASNase activity was measured as per Shifrin et al. [29]. We prepared a stock solution (1 mg/mL) through dissolving 0.1 g of the free or prepared nano ASNase powder in 100 mL potassium phosphate buffer (0.05 M, pH 8.0). The reaction mixture comprised 1 mL of prepared stock enzyme (1 mg/mL), 2 mL of 20 mM L-asparagine, and 2 mL of potassium phosphate buffer (0.05 M, pH 8.0). After gentle mixing and incubation at 37 °C for 30 min, the reaction was stopped with 1 mL of 1.5 M trichloroacetic acid (TCA), followed by centrifugation at 15,000 rpm for 10 min to remove precipitates. Ammonia concentration was determined using Nessler’s reagent. We added 1 mL of Nessler’s reagent to 1 mL of the resulted enzyme reaction supernatant and left it for 10 min at room temperature to develop the color (yellow to brownish orange). The blank was prepared by adding the enzyme post-TCA addition, and the resulting color was measured at 436 nm. A calibration curve (Supplementary data Figure S1) was established by preparing a series of known ammonia concentrations in the same reaction mixture. One unit of L-ASNase activity was defined as the amount of enzyme releasing 1 µmol of ammonia per minute under these conditions.

#### 2.2.2. Circular Dichroism Spectroscopy (CD)

In this study, CD measurements were performed to assess the secondary structure of both free and nano-prepared ASNase. The CD spectra were collected to detect any conformational changes or stability differences between the free enzyme and the nanoformulated version. Measurements were taken using a 0.1 cm path length quartz cuvette containing 1 mg/mL ASNase. The cuvette was filled with a stock solution prepared by dissolving 0.1 g of either free or nano-prepared ASNase in 100 mL of potassium phosphate buffer (0.05 M, pH 8.0). The secondary structure analysis focused on identifying and quantifying elements such as alpha-helices, beta-sheets, and random coils. This was achieved by averaging eight scans per sample over the wavelength range of 190 to 260 nm using a Jasco J-810 spectropolarimeter (Easton, MD, USA) at 25 °C, followed by computational analysis with CDNN 2.1 software (ACGT Progenomics AG, Halle, Germany).

#### 2.2.3. Effect of pH and Temperature on ASNase Activity and Stability

The optimum pH for both free and immobilized ASNase activity was determined by assaying the enzyme at various pH levels at 30 °C (The optimum temperature for the tested form of ASNase enzyme). Enzyme stability under different storage pH conditions was assessed after storing the enzyme in various buffers for 24 h. Enzyme activity was then measured, with stability expressed as relative activity compared to that of fresh enzyme (non-stored free or immobilized ASNase was considered as being 100%). The optimum temperature for both free and immobilized ASNase activity was found by measuring enzyme activity at different temperatures (15–75 °C) at pH 8.5. Enzyme stability at different storage temperatures (15–75 °C) was similarly assessed after a 24 h storage period; stability was, again, expressed as relative activity compared to that of the fresh enzyme (non-stored free or immobilized ASNase was considered as being 100%).

#### 2.2.4. Kinetic Parameters

Kinetic parameters, including the Michaelis constant (K_m_) and maximum velocity (V_max_, reaction rate at which the substrate saturates all enzyme sites), were determined using Lineweaver-Burk plots with L-asparagine as substrate in concentrations ranging from 2 to 40 mM. Following enzyme assay protocols with 0.5 mg enzyme in the assay mixture, activity was determined for each substrate concentration. According to Egbune et al. [30], K_m_, V_max_, and the turnover number (K_cat_) were calculated as follows:

Vertical intercept = 1/V_max_ (2)

Horizontal intercept = −1/K_m_ (3)

Kcat = V_max_/[E]_t_ (4)

where [E]_t_ is the total molar concentration of enzyme found in the reaction mixture equal to the amount of used enzyme/enzyme molecular weight.

At the same time, the quantities called enzyme catalytic efficiency and enzyme specificity constant were defined as follows:

Enzyme catalytic efficiency = K_cat_/K_m_ (5)

Enzyme specificity constant = V_max_/K_m_ (6)

The ratio V_max_/K_m_ serves as an indicator of an enzyme’s apparent specificity for a substrate under the given reaction conditions, though it does not reflect intrinsic catalytic efficiency or activity, as it depends on enzyme concentration. In contrast, K_cat_/K_m_ provides a more accurate measure of catalytic efficiency, as it represents how effectively an enzyme converts substrate to product, particularly at low substrate concentrations [31].

#### 2.2.5. Enzyme Shelf Life

The activity of both free and immobilized ASNase was measured after storage at room temperature for various periods. Activity of the fresh enzyme was set as 100%.

#### 2.2.6. Application of ASNase for L-Asparagine Hydrolysis in Human Serum

To evaluate the feasibility of using immobilized ASNase in vivo as cancer treatment, activity tests were conducted in a commercial human serum medium, which mimics the complex mixture of human serum. L-asparagine was added to the serum, as it does not naturally contain L-asparagine, and the hydrolysis by both immobilized and free enzymes was compared as described by Orhan and Uygun [32].

### 2.3. Anticancer Activity of ASNase

#### 2.3.1. Cell Viability Test

The anticancer activity of free and immobilized ASNase was assessed using the MTT assay. The human cancer cell lines used included lung carcinoma (A549), breast adenocarcinoma (MCF7), colon carcinoma (HCT 116), and leukemic T-cell lymphoblast (Jurkat E6.1) obtained from Sigma Aldrich. Anticancer activity was determined based on the IC_50_ (half-maximal inhibitory concentration) values of the enzymes [33].

#### 2.3.2. High-Content Screening Assay

A high-content screening assay evaluated toxicity in various cancer cells, examining parameters like nuclear intensity, membrane permeability, mitochondrial membrane permeability, and cytochrome c release. Cells were seeded at 10^5^ per well in 12-well plates and incubated at 37 °C with 5% CO_2_ for 24 h. Cells were then treated with IC50 concentrations of different enzyme preparations, alongside untreated controls and cells treated with 1 mM doxorubicin (positive control). After another 24 h incubation, MMP dye (MitoTracker™ Orange CMTMRos, Excitation 552/Emission 572) and cell permeability dye (BioTracker™ 490 Green Cytoplasmic Membrane Dye, Excitation 491/Emission 509) were added, followed by a 1 h incubation. Cells were fixed with 4% formaldehyde for 15 min, permeabilized with 0.1% Triton X-100 in PBS, and blocked with 3% bovine serum albumin. Cytochrome c was stained using a primary mouse antibody for 1 h, followed by washing and staining with goat anti-mouse secondary antibodies conjugated with DyLight™ 649. Nuclei were stained with Hoechst 33258. Visualization and quantification of fluorescence intensity were performed using the Cellomics ArrayScan high-content screening reader (Thermo Scientific, Waltham, MA, USA) and the Cell Health Profiling Bioapplication module [34]. All chemicals used are obtained from Sigma Aldrich except MitoTracker™ Orange CMTMRos and DyLight™ 649 are obtained from Thermo Fisher Scientific

##### Statistical Analysis

The statistical analysis was performed by SPSS software (version 14). Data were expressed as mean ± standard error. A comparison of the mean between different ASNase was performed using ANOVA test, *p* < 0.01.

## 3. Results

### 3.1. Immobilization Efficiency on Different Types of Nanoparticles

**Figure 1:**
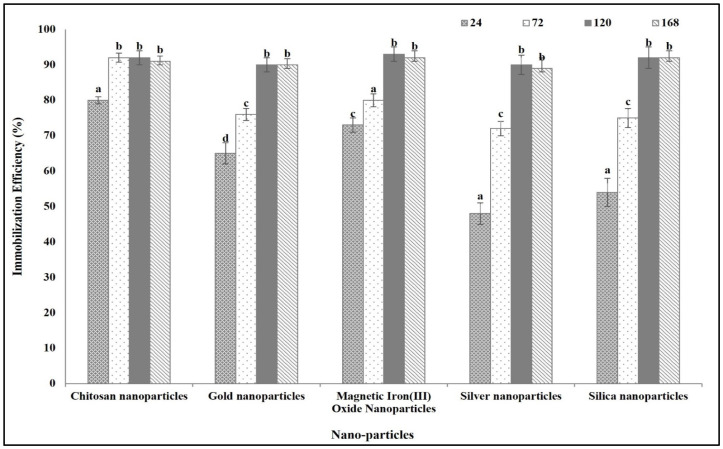
*E. coli* ASNase immobilization efficiency on different studied nanoparticles after different incubation periods (h). Columns values are mean of three replicates. Error bars represent standard deviations. Columns followed by different letters are significantly different from each other according to ANOVA test, *p* < 0.01.

The highest immobilization efficiency recorded in all studied nanoparticles was 90%. The difference between the studied nanoparticles was in the time at which this maximum efficiency was recorded. Chitosan nanoparticles were the fastest in reaching the maximum immobilization efficiency (90%) after 72 h. On the other hand, the other sorts of nanoparticle used, spherical gold nanoparticles, silver nanoparticles, magnetic iron(III) oxide nanoparticles, and mesoporous silica nanoparticles, reached the maximum immobilization efficiency after 120 h (Figure 1).

### 3.2. Effect of Immobilization on Different Nanoparticles on ASNase Characters

#### 3.2.1. Effect on ASNase Secondary Structure

**Figure 2:**
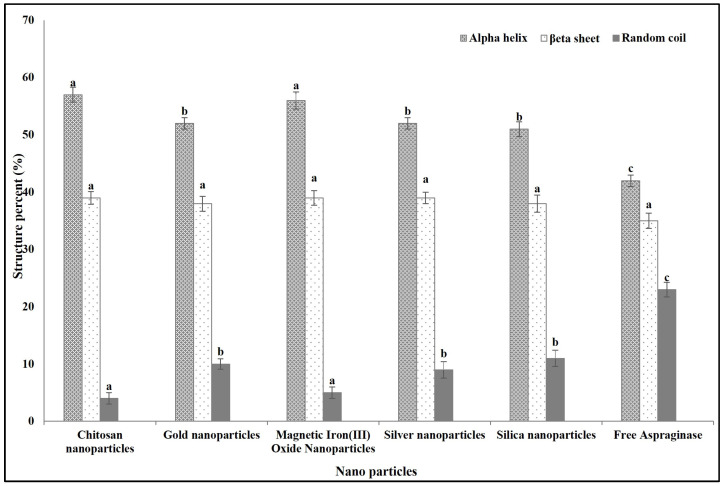
Effect of immobilization on different studied types of nanoparticles on the *E. coli* ASNase secondary structures percentages at 25 °C. Columns values are mean of three replicates. Error bars represent standard deviations. Columns for the same structure followed by different letters are significantly differ from each other according to ANOVA test, *p* < 0.01.

We used a 72 h incubation period to obtain ASNase immobilized on chitosan. For other types of nanoparticles, spherical gold nanoparticles, silver nanoparticles, magnetic iron(III) oxide nanoparticles, and mesoporous silica nanoparticles, a 120 h incubation period was used for immobilization. The immobilization of ASNase on different types of nanoparticles caused differences in its secondary structures compared to the free one. Immobilization on all studied nanoparticles induced a significant decrease in the random coil percentage and a significant increase in the alpha helix percentage, while there was no significant change in the beta sheet content. Interestingly, immobilization onto chitosan and magnetic iron(III) oxide caused a significant increase in the alpha helix percentage and a significant decrease in the random coils percentage compared to immobilization on the other studied types of nanoparticles (Figure 2).

#### 3.2.2. Effect of Temperature on Free and Immobilized ASNase Activity and Stability

**Figure 3:**
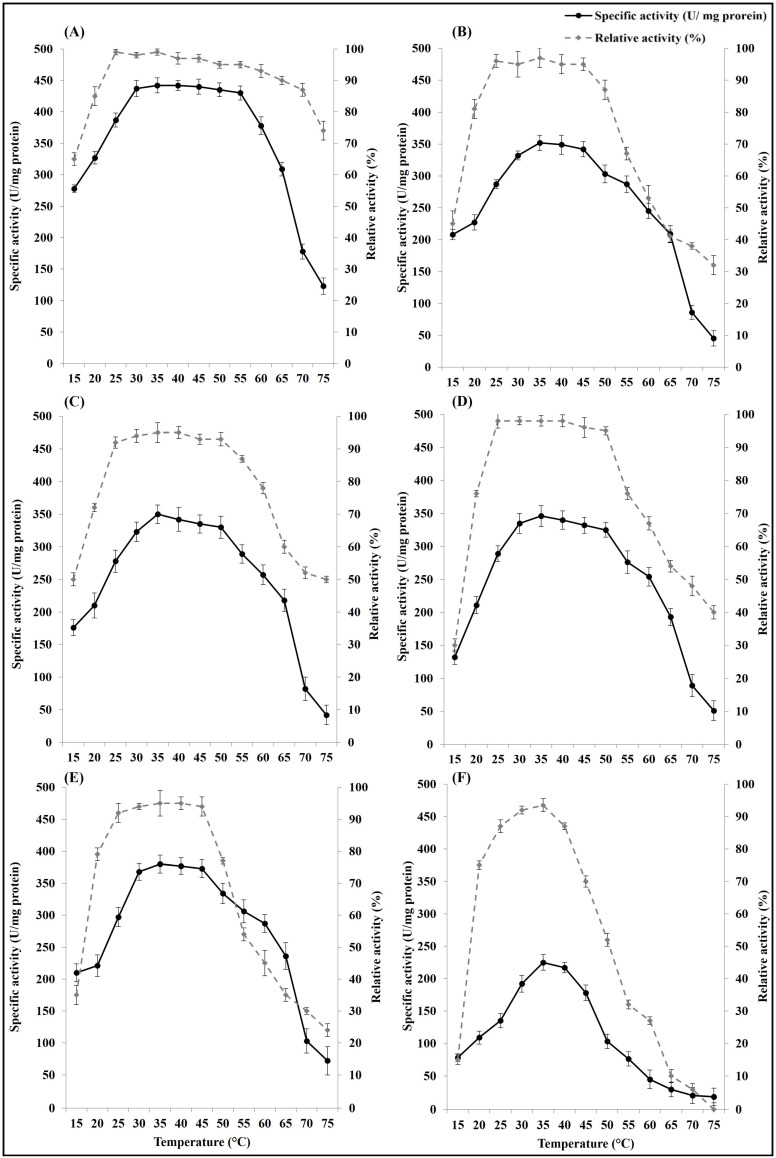
Effect of temperature on activity and stability (relative activity after enzyme incubation for 24 h at different temperatures) of free *E. coli* ASNase (**F**) and *E. coli* ASNase immobilized on different types of nanoparticles: (**A**) chitosan nanoparticles, (**B**) gold nanoparticles, (**C**) silver nanoparticles, (**D**) silica nanoparticles, and (**E**) magnetic iron(III) oxide nanoparticles. Plotted values taken individually are an average of three replicates. Error bars represent standard deviations.

Immobilization on the five types of nanoparticles improved the storage stability and the activity of ASNase under different temperature changes. The optimum temperature range regarding the highest activity of the free ASNase was 35–40 °C, while after the enzyme immobilization, the optimum temperature range was 30–55 °C in case of immobilization on nano chitosan and 30–45/50 °C in case of immobilization on the other studied nanoparticles. Also, immobilization increased the temperature storage stability range at which ASNase can retain more than 90% of its activity after incubation at different temperature for 24 h. The widest stability temperature range was recorded in the ASNase immobilized on nano chitosan (25–65 °C), followed by ASNase immobilized on silver nanoparticles (25–50 °C), and then ASNase immobilized on the other types of nanoparticles (25–45 °C), while it was from 30 to 35 °C in the free ASNase (Figure 3).

#### 3.2.3. Effect of pH on Free and Immobilized ASNase Activity and Stability

The changes in pH over the optimum pH significantly decreased the ASNase activity. The optimum pH of the free ASNase was between 8.5 and 9. The optimum pH range became wider after immobilization on nanoparticles. The widest optimum pH range was recorded in the ASNase immobilized on chitosan (7.5–10.5), followed by that found in the enzyme immobilized on nanoparticles of silica (7.5–10), magnetic iron(III) oxide (7–9.5), gold (8–10), and silver (7.5–9.5).

**Figure 4:**
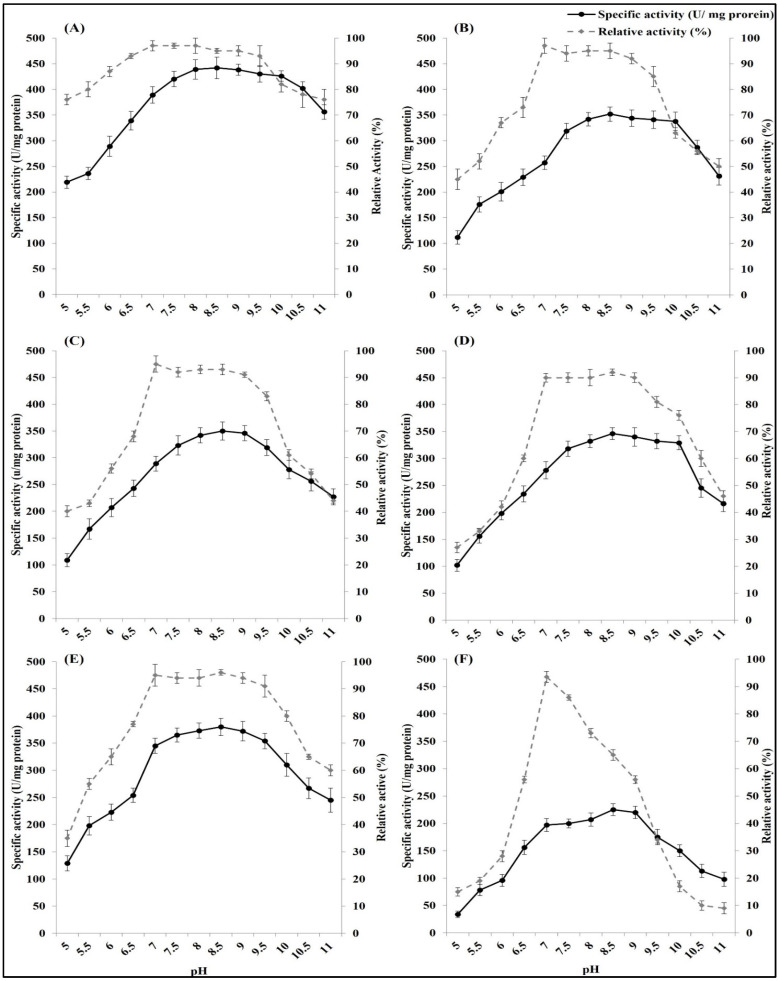
Effect of pH on activity and stability (relative activity after enzyme incubation for 24 h at different pH) of free *E. coli* ASNase (**F**) and *E. coli* ASNase immobilized on different types of nanoparticles: (**A**) chitosan nanoparticles, (**B**) gold nanoparticles, (**C**) silver nanoparticles, (**D**) silica nanoparticles, and (**E**) magnetic iron(III) oxide nanoparticles. Plotted values taken individually are an average of three replicates. Error bars represent standard deviations.

Similarly, the pH storage stability range of *E. coli* ASNase increased through immobilization on nanoparticles. The free ASNase showed a single pH point (pH 7) at which the enzyme can be incubated for 24 h and retain more than 90% of its original activity, and away from this point, the free ASNase substantially lost its activity after incubation. ASNase immobilization on chitosan led to the widest pH stability range (6.5–9.5), followed by immobilization on nano magnetic iron (III) oxide (7–9.5). Meanwhile, ASNase immobilized on silver, silica, and gold had a pH stability range from 7 to 9 (Figure 4).

#### 3.2.4. Kinetics Parameters of Free and Immobilized ASNase Activity and Stability

**Figure 5:**
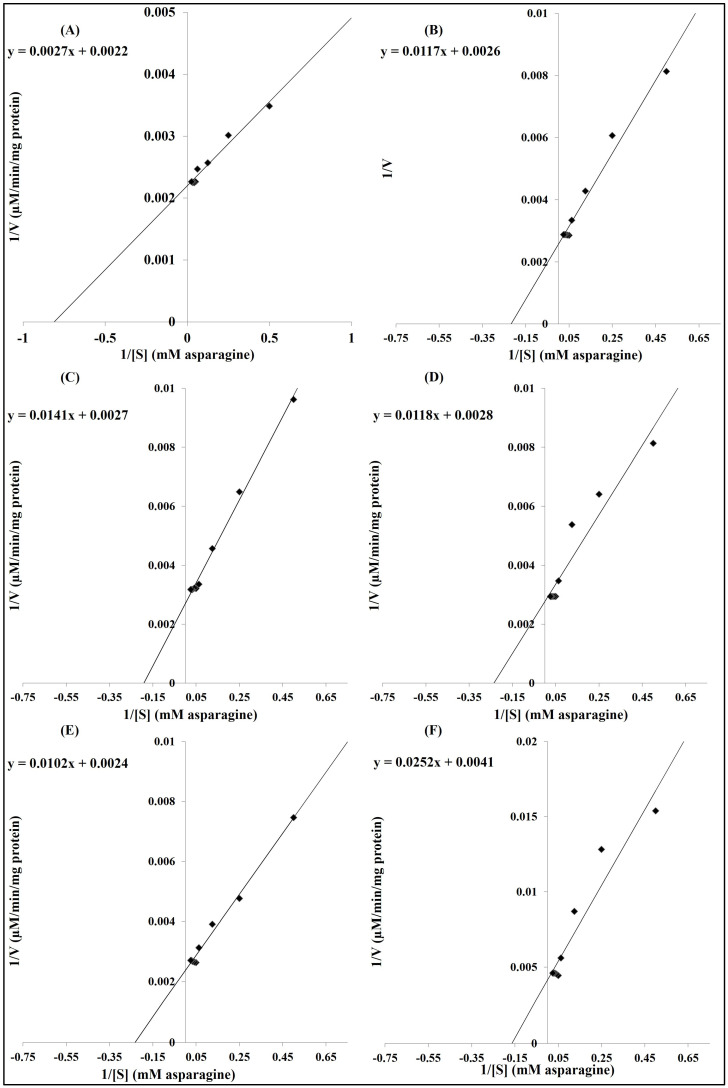
Lineweaver–Burk plots curve relating to free ASNase (**F**) and ASNase immobilized on different types of nanoparticles: (**A**) chitosan nanoparticles, (**B**) gold nanoparticles, (**C**) silver nanoparticles, (**D**) silica nanoparticles, and (**E**) magnetic iron (III) oxide nanoparticles.

Enzyme kinetics provides insights into the molecular mechanisms by which enzymes catalyze reactions by examining the rates of enzyme-catalyzed reactions. The results in Table 1 and Figure 5 show the kinetics of free and immobilized ASNase on studied nanoparticles. Generally, ASNase kinetics significantly improved through immobilization on nanoparticles. The maximum reaction rate (V_max_) was significantly higher in the immobilized ASNase than in the free ASNase. The V_max_ values of ASNase immobilized on nano chitosan and ASNase immobilized on nano magnetic iron (III) oxidewere non-significantly different, but they were higher than those of the other studied systems. The Michaelis–Menten constant (K_m_) was the highest in the free ASNase (6.15 mM asparagine) and the lowest in the nano-chitosan-immobilized ASNase (1.23 mM asparagine). Nano-gold ASNase, magnetic-iron(III)-oxide-immobilized ASNase, and nano-silica-immobilized ASNase had displayed k_m_ values of 4.50, 4.25, and 4.21 mM asparagine, respectively; these values were non-significantly different from each other, but significantly lower than that of the free ASNase and significantly higher than that of the nano-chitosan-immobilized ASNase. In the same line, the other kinetics parameters, namely, the turnover number (K_cat_), enzyme catalytic efficiency (K_cat_/K_m_), and enzyme specificity constant = V_max_/K_m_ were significantly higher in the immobilized ASNase than those for the free enzyme. The highest values were found in the nano-chitosan-immobilized ASNase, followed by, first, the nano-magnetic-iron(III)-oxide-immobilized ASNase, and then, by nano-gold-immobilized ASNase, nano-silver-immobilized ASNase, and nano-silica-immobilized ASNase.

#### 3.2.5. Free and Immobilized ASNase Shelf Life Time

**Figure 6:**
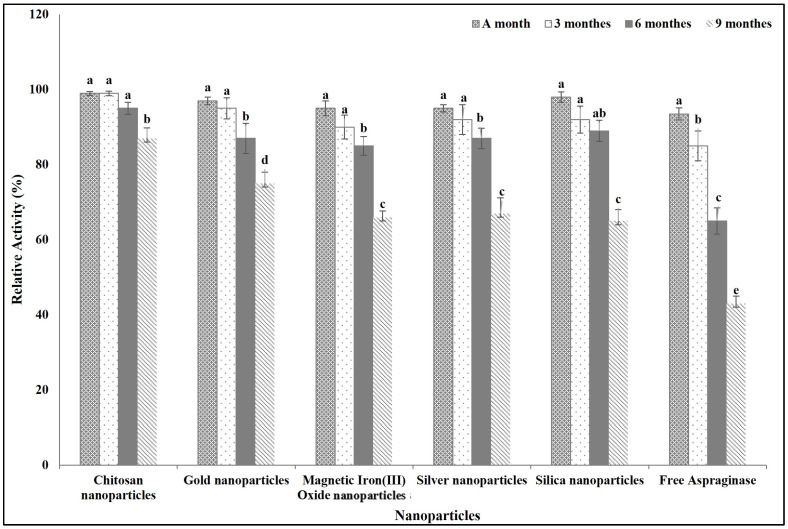
Effect of storage time on free and immobilized ASNase activity. Columns values are mean of three replicates. Error bars represent standard deviations. Columns for the same time followed by different letters are significantly differ from each other according to ANOVA test, *p* < 0.01.

Figure 6 shows the effect of different incubation times on the activity of free and immobilized ASNase. The nano-chitosan-immobilized ASNase showed the highest ability to retain its activity over the time. The enzyme retained more than 90% of its activity after incubation for 6 months at room temperature and it retained more than 85% of its original activity after incubation for 9 months at the same temperature, while the other immobilized ASNase kept more than 65% of its original activity after incubation for 9 months at room temperature. On the other hand, the free ASNase preserved only 43% of its activity at the same incubation conditions.

#### 3.2.6. Application of Free and Immobilized ASNase for L-Asparagine Hydrolysis in Human Serum

**Figure 7:**
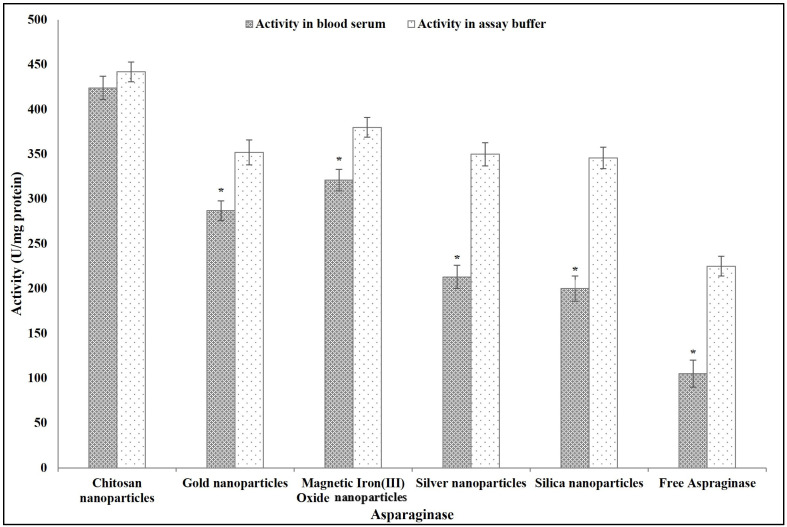
The feasibility of using free and immobilized ASNase in vivo as cancer treatment assessed by activity tests conducted in a commercial human serum medium. Columns values are mean of three replicates. Error bars represent standard deviations. Columns followed by asterisk are significantly differ from activity in the assay buffer according to paired T test, *p* < 0.01.

The results in Figure 7 show that the activity of ASNase in the blood serum was significantly lower than that in the assay buffer, except for ASNase immobilized on chitosan. The activity of the nano-chitosan-immobilized ASNase in the blood serum was non-significantly different from that in assay buffer. The activity values of other immobilized ASNase (nano-gold-immobilized ASNase, nano-magnetic-iron(III)-oxide-immobilized ASNase, nano-silver-immobilized ASNase, and nano-silica-immobilized ASNase) in the blood serum were significantly lower than those determined in the assay buffer (19, 16, 39, and 42%, respectively). The free ASNase activity decreased in the blood serum by 53% compared to that measured in the assay buffer.

### 3.3. Anticancer Activity of ASNase

#### 3.3.1. IC_50_ of Free and Immobilized ASNase

**Figure 8:**
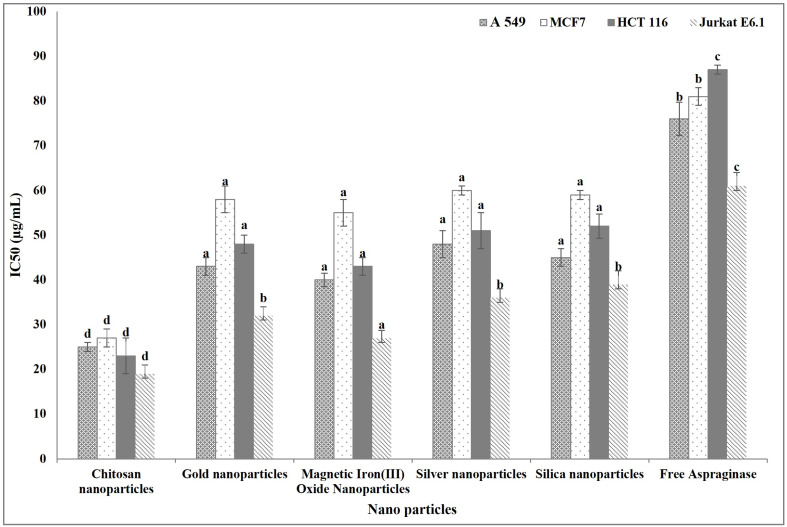
Anticancer activity (IC_50_) of free and immobilized ASNase. Columns values are mean of three replicates. Error bars represent standard deviations. Columns for the same cancer cell line followed by different letters are significantly differ from each other according to ANOVA test, *p* < 0.01.

The anticancer activity of free and immobilized ASNase was evaluated through their IC_50_ against different human cancer cell lines. The ASNase immobilized on different nanoparticles had an IC_50_ significantly lower than that of the free ASNase. Interestingly, nano-chitosan-immobilized ASNase had the lowest IC_50_ and there were non-significant differences in the IC_50_ among the ASNase immobilized on the other nanoparticles (Figure 8).

#### 3.3.2. Anticancer Activity Mechanism (High-Content Screening Assay)

**Figure 9:**
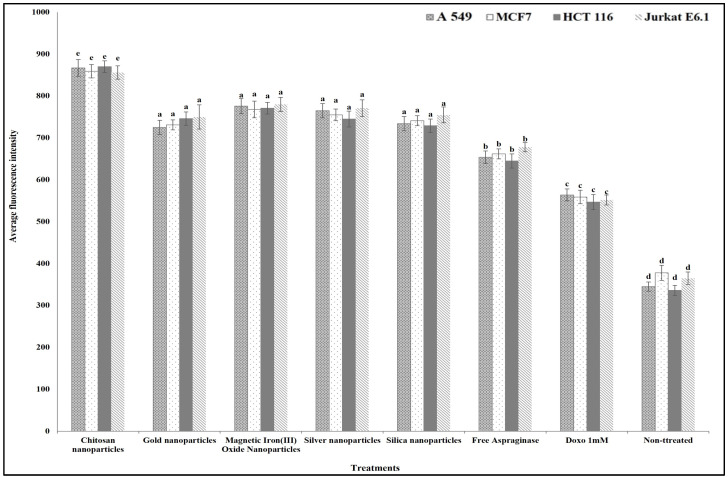
The effect of free and immobilized ASNase on nuclear intensity release in different cancer cell lines. Columns values are mean of three replicates. Error bars represent standard deviations. Columns for the same cancer cell line followed by different letters are significantly differ from each other according to ANOVA test, *p* < 0.01.

**Figure 10:**
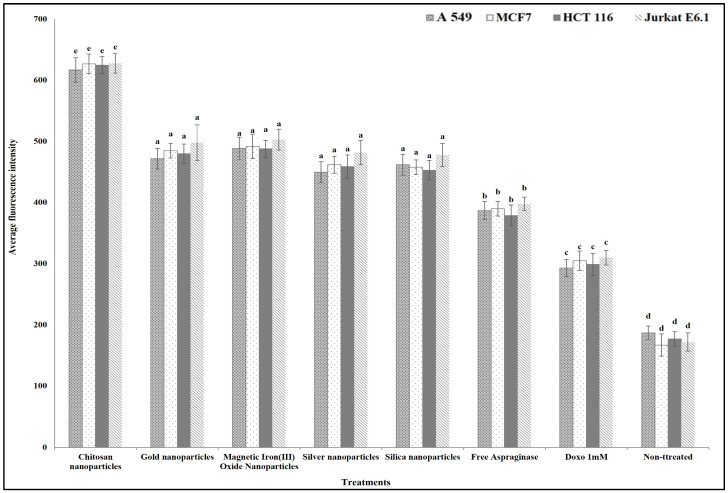
The effect of free and immobilized ASNase on plasma membrane permeability in different cancer cell lines. Columns values are mean of three replicates. Error bars represent standard deviations. Columns for the same cancer cell line followed by different letters are significantly differ from each other according to ANOVA test, *p* < 0.01.

**Figure 11:**
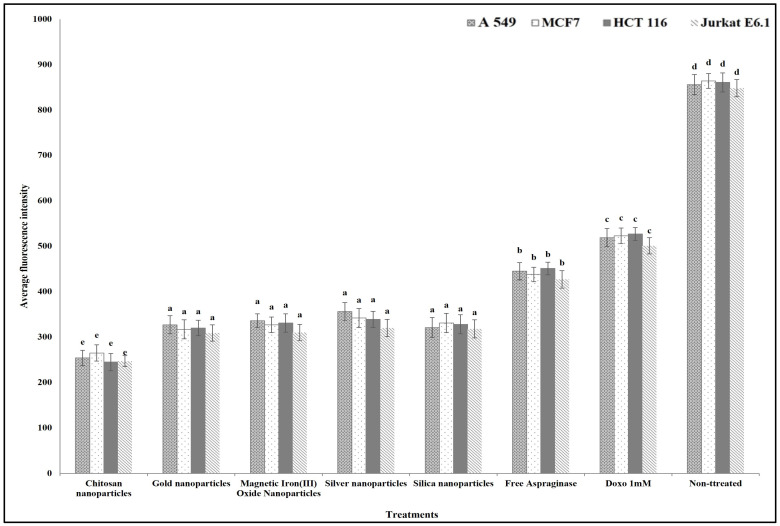
The effect of free and immobilized ASNase on mitochondrial membrane permeability in different cancer cell lines. Columns values are mean of three replicates. Error bars represent standard deviations. Columns for the same cancer cell line followed by different letters are significantly differ from each other according to ANOVA test, *p* < 0.01.

**Figure 12:**
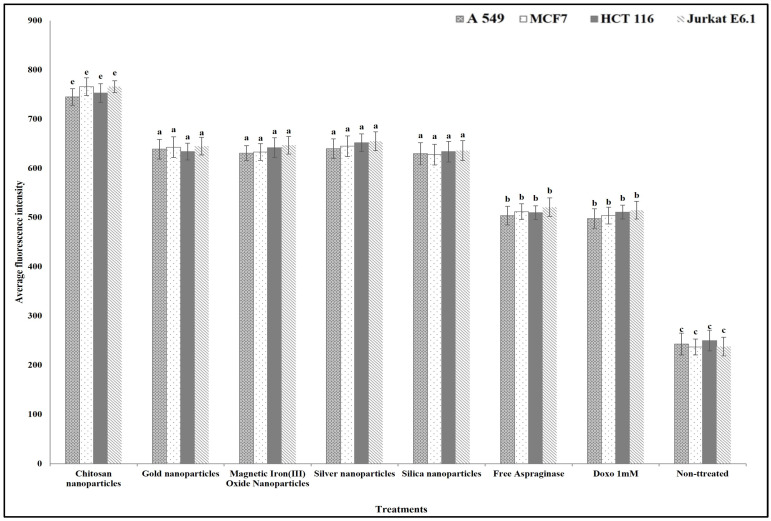
The effect of free and immobilized ASNase on cytochrome release in different cancer cell lines. Columns values are mean of three replicates. Error bars represent standard deviations. Columns for the same cancer cell line followed by different letters are significantly differ from each other according to ANOVA test, *p* < 0.01.

For studying the anticancer mechanism by which ASNase can destroy the cancer cells, we determined the effect of free and different immobilized ASNase treatment on the cancer cells’ plasma membrane permeability, nuclear intensity, mitochondrial membrane permeability, and cytochrome release in comparison with standard anticancer agent doxorubicin (positive control). The results show that different free and immobilized ASNase systems were significantly more effective than doxorubicin in increasing cancer cells` nuclear intensity (Figure 9), plasma membrane permeability (Figure 10), mitochondrial membrane permeability (Figure 11), and cytochrome release (Figure 12). Comparatively, free ASNase was significantly less effective than that immobilized onto the nanoparticles studied, these systems being non-significantly different from each other in their effect.

## 4. Discussion

Enzymes are employed in cancer therapy through various innovative strategies, including enzyme prodrug therapy, the direct enzymatic degradation of tumor components, the enhancement of drug delivery, and the modulation of the immune response. These approaches highlight the versatility and potential of enzymes as powerful tools in the fight against cancer [16,35]. Therapeutic enzyme immobilization enhances the practicality, efficiency, and safety of enzyme-based treatments, making it a critical technique in modern medical and biotechnological applications [36,37,38]. Choosing materials for efficient enzyme immobilization is an important point in their applications. The time needed for reaching the maximum enzyme immobilization under normal conditions is an important factor for choosing the immobilization materials [39,40,41]. The present results showed that chitosan nanoparticles were the fastest to reach the maximum immobilization efficiency of ASNase. The enzyme immobilization process on nano chitosan is faster than on the other nanoparticles studied in this work due to several factors. Chitosan nanoparticles possess polar amino and hydroxyl groups active in inducing attractive interactions chitosan enzymes. This aspect may explain the quicker attachment of the enzymes/proteins to the chitosan nanoparticles surface compared to that of metal/metal-containing nanoparticles, which may require additional surface modifications to achieve similar affinity [42,43,44]. In acidic environments (pH < 5.5), the cationic nature of chitosan, due to its protonated amino groups (-NH^3+^), can facilitate strong electrostatic interactions with negatively charged enzyme molecules, enhancing the rate of enzyme attachment to chitosan nanoparticles. At neutral and basic pH, however, chitosan’s amino groups are largely unprotonated (-NH_2_), which diminishes these interactions. The extent of this attraction also depends on the isoelectric point of ASNase and the pH of the environment [45,46]. Chitosan nanoparticles often have a porous structure with a high surface area, providing more binding sites for enzymes. The porous nature allows enzymes to penetrate and attach quickly, unlike with some metal nanoparticles, which may have a more compact and less accessible surface [42,44,47].

Enzyme immobilization on nanoparticles can significantly improve their structure and characteristics in several ways [48]. The present results showed that immobilization on nanoparticles improved the secondary structure of ASNase through decreasing the random coil percent. Decreasing the random coil content in the enzyme structure enhances its stability and in turn its activity [49]. The nanoparticles provide a scaffold that maintains the enzymes in a more favorable conformation. The large surface area and high surface energy of nanoparticles provide extensive binding sites for enzymes, promoting correct folding and the stabilization of secondary structures such as α-helices and β-sheets [50]. So, hydrogen bonds, van der Waals forces, and hydrophobic interactions between the enzyme and the nanoparticle surface can stabilize specific secondary structures, reducing the tendency of the enzyme to adopt random coil folding [51]. Binding to nanoparticles restricts the conformational freedom of the enzyme. This restriction can prevent the enzyme from adopting less stable, random coil conformations, favoring more stable α-helix and β-sheet structures. Immobilization often involves multiple attachment points, which help maintain the enzyme in a more rigid and ordered conformation, minimizing the presence of random coils [52,53,54].

Immobilized enzymes often exhibit increased thermal stability compared to their free counterparts [55]. Similarly, our results indicated that immobilized ASNase on nanoparticles had a significantly higher temperature activity/stability range than free ASNase. This is related to the attachment to nanoparticles, which restricts the enzyme’s conformational flexibility, making it less susceptible to denaturation at higher temperatures [56,57]. The immobilization materials can act as thermal buffers, absorbing and dissipating heat, thereby protecting the overall enzyme structure from rapid temperature changes [58].

Immobilized enzymes are typically more resistant to changes in pH [59]. Similarly, our results indicated that immobilized ASNase on different types of nanoparticles had a wider pH activity/stability range than the free ASNase. Immobilization increases the resistance of enzymes to the changes in pH, as the immobilization support can create a microenvironment around the enzyme that buffers against pH changes. This local environment can help maintain the enzyme’s optimal pH range, even when the external pH fluctuates [60,61]. Thus, immobilization can limit the diffusion of hydrogen or hydroxide ions to the enzyme, thereby reducing the impact of pH changes on enzyme activity [59].

Immobilization also improves the enzyme kinetics parameters for more efficient catalytic performance [62]. The results of the present study revealed the positive role played by immobilization in improving the kinetics parameters of the ASNase such as K_m_ (Michaelis constant), V_max_ (maximum velocity), and K_cat_ (turnover number). This is because immobilization can increase the thermal, chemical, and operational stability of enzymes. Such a stabilization helps maintain the enzyme’s active conformation, leading to higher catalytic efficiency (K_cat_/K_m_) and maximum velocity (V_max_) [16,59]. Also, the high surface area of nanoparticles provides a large number of binding sites for enzyme molecules, which can lead to higher enzyme loading and increased reaction rates [36,63]. Immobilization improves the enzyme configuration that enhances the binding and activity of the immobilized enzyme, and this improves the catalytic efficiency (K_cat_/K_m_) and enzyme specificity constant (V_max_/K_m_) [41,54].

Immobilization extends enzyme shelf life and makes enzymes more suitable for industrial and biotechnological applications by providing greater stability and activity under different conditions [36,59]. Similarly, our results indicated that the improvement in the structure, activity, and stability of ASNase through immobilization resulted in a longer shelf time for the immobilized ASNase than for the free one.

ASNase is a well-known therapeutic enzyme used in cancer therapy. The activity of the ASNase in a biological medium such as blood serum is important for its usability. The immobilization of ASNase on different types of nanoparticles enhanced its activity in blood serum by comparison with the same property measured in its non-bound/free state. Similarly, Orhan and Uygun [32] reported the efficiency of immobilization in saving the ASNase activity in the blood serum. This is related to the efficiency of immobilization to form a shield that can protect the enzyme from different unfavorable conditions [51]

The improvement of the structure, stability, and activity of ASNase through immobilization on nanoparticles was noticed in its anticancer activity against different cancer cell lines used in this study. The IC_50_ values of the *E. coli* ASNase immobilized on different types of nanoparticles were lower than those of the free enzyme, and so the anticancer activity of the ASNase in the immobilized state was higher than that of the enzyme in its free state. In line with that, Iraninasab et al. [18] reported the superiority of the immobilized ASNase compared to its free state as an anticancer agent against human acute lymphoblastic leukemia. Also, the immobilized ASNase was found to be effective against chronic myeloid leukemia compared to the free enzyme [64]. In this paper, the immobilized ASNase caused a significantly negative effect on the studied apoptosis markers (cancer cells nuclear intensity, membrane permeability, mitochondrial membrane permeability, and cytochrome c release) by comparison with doxorubicin. These markers are considered a useful set of parameters by which cancer cell apoptosis can be evaluated [65]. The main action of L-ASNase in catalyzing the conversion of L-asparagine (an important amino acid synthesized by normal cells but not by cancer cells) leads to the depletion of L-asparagine in cancer cells, disrupting cellular signaling pathways and, eventually, resulting in cancer cells apoptosis [22,23,24,25].

Our results proved that the ASNase immobilized on nano chitosan was more effective in improving enzyme stability, activity, specificity, and usability as an anticancer agent compared to immobilization on the other metal and metal-containing nanoparticles studied (Supplementary data Table S6). In acidic environments (pH < 5.5), the cationic nature of chitosan, due to its protonated amino groups (-NH^3+^), can facilitate strong electrostatic interactions with negatively charged enzyme molecules, enhancing the rate of enzyme attachment to chitosan nanoparticles. At neutral and basic pHs, however, chitosan’s amino groups are largely unprotonated (-NH_2_), which diminishes these interactions. The extent of this attraction also depends on the isoelectric point of ASNase and the pH of the environment [45,46]. The content of amino groups and hydroxyl groups in the structure of chitosan nanoparticles promotes the formation of ionic interactions and hydrogen bonds with amino acid side substituents in the enzyme structure [66]. Functionalizing chitosan with specific groups can orient enzymes favorably for optimal substrate interaction that is often not achievable with metal nanoparticles. [67]. Also, the porous structure of nano chitosan helps protect enzymes from environmental stress and enhances the mass transfer of substrates and products, leading to improved enzyme activity and efficiency [47]. The hydrophilic nature of nano chitosan can provide a favorable environment for enhancing enzyme activity, as enzymes typically perform better in aqueous conditions [68,69].

## 5. Conclusions

In conclusion, enzyme immobilization on nanoparticles, particularly, nano chitosan, has shown significant promise in enhancing enzyme stability, activity, and therapeutic potential, especially in cancer therapy. Chitosan nanoparticles, with their abundant functional groups and porous structure, facilitate efficient enzyme attachment and promote a favorable environment for enzymatic reactions. Compared to other nanoparticles, chitosan nanoparticles provide superior results in terms of enzyme stabilization, improving ASNase’s structural integrity, thermal and pH stability, and catalytic efficiency. The immobilized ASNase demonstrated enhanced anticancer activity, as reflected in its lower IC_50_ and stronger induction of cancer cell apoptosis compared to its free form. These findings highlight the potential of nano chitosan as a superior immobilization material, offering a powerful tool for optimizing enzyme-based therapies and expanding their application in the medical and biotechnological fields. Further studies are needed to evaluate the immobilization yield and to conduct additional structural characterization of enzymes post-immobilization.

## Figures and Tables

**Table 1:** Free and immobilized ASNase kinetic parameters.

	Parameter	K_m_ (mM Asparagine)	Vm_ax_ (µM/min/mg Protein)	K_cat_ (s^−1^)	V_max_/K_m_ (µM/min/mg Protein)/mM)	K_ca_t/K_m_ (mM^−1^⋅s^−1^)
Enzyme						
Nano-Chitosan-Immobilized ASNase		1.23 ± 0.152 a	454.55 ± 20.67 a	61960.88 a	369.55 a	50497.86 a
Nano-Gold-Immobilized ASNase		4.50 ± 0.144 b	384.62 ± 16.90 b	52428.44 b	85.47 b	11650.76 b
Nano-Magnetic-Iron(III)-Oxide-Immobilized ASNase		4.25 ± 0.096 b	416.67 ± 21.49 a	56797.57 ab	98.04 c	13364.13 c
Nano-Silver-Immobilized ASNase		5.22 ± 0.151 c	370.37 ± 19.87 b	50486.64 b	70.95 d	9671.77 d
Nano-Silica-Immobilized ASNase		4.21 ± 0.078 b	357.14 ± 20.89 b	48683.62 b	84.83 b	11563.81 b
Free ASNase		6.15 ± 0.137 d	243.90 ± 15.78 c	33247.27 c	39.66 e	5406.06 e

Values are mean of three replicates ± standard deviation. Values in the same column followed by different letters are significantly different from each other according to ANOVA test, *p* > 0.001.

## Data Availability

All data generated in this study are found in this manuscript.

## Supplementary Materials

The following supporting information can be downloaded at: https://www.mdpi.com/article/10.3390/polym16233260/s1, Figure S1: Ammonia calibration curve using Nessler’s reaction; Table S1: Chitosan Nanoparticles Description NCZ-MN-116/20 Description; Table S2: Spherical Gold Nanoparticles (NCZ-ST-192/22) Description; Table S3: Silver nanoparticles (NCZ-ST-271/22) Description; Table S4: Magnetic Nanoparticles Iron III Oxide Ferrosoferric Oxide (NCZ- ST-128/23) Description; Table S5: Mesoporous silica nanoparticles (NCZ-NP-620/24) Description; Table S6: Relative enzyme activity.
