# Supplementary material for: Enhancing the Stability and Anticancer Activity of Escherichia coli Asparaginase Through Nanoparticle Immobilization: A Biotechnological Perspective on Nano Chitosan
Source: Polymers (Basel). 2024 Nov 23;16(23):3260. doi: 10.3390/polym16233260 (PMC11644295; doi:10.3390/polym16233260)
Supplement: Supplementary file 1 [file polymers-16-03260-s001.zip › polymers-3260442-supplementary.pdf]

Table S1: Chitosan Nanoparticles Description NCZ-MN-116/20 Description

Product	**Chitosan Nanoparticles**
Cat No	NCZ-MN-116/20
CAS	9012-76-4
Purity	>99.9%
APS	80-100nm
Molecular Formula	C6H11NO4

Table S2: Spherical Gold Nanoparticles (NCZ-ST-192/22) Description

**Product**	**Spherical Gold Nanoparticles (90 nm)**
CASNo.	7440-57-5
Purity	≥99.9%
APS	90 nm
Chemical Formula	AU
Product Code	NCZ-ST-192/22

Table S3: Silver nanoparticles (NCZ-ST-271/22) Description

Product Name	**Silver nanoparticles**
CASNo.	7440-22-4
Purity	≥99.9%
APS	80 nm
Chemical Formula	Ag
Product Code	NCZ-ST-271/22

Table S4:　Magnetic Nanoparticles Iron III Oxide Ferrosoferric Oxide (NCZ-ST-128/23) Description

Product Name	**Magnetic Nanoparticles Iron III Oxide Ferrosoferric Oxide**
CAS No.	1332-37-2
Purity	≥99.9%
APS	20 - 100 nm
Ingredient	Fe_2O_3
Product Code	NCZ- ST-128/23

Table S5: Mesoporous silica nanoparticles (NCZ-NP-620/24) Description

Product	**Mesoporous Silica Nanoparticles**
CAS No.	7631-86-9
Purity	≥99%
APS	80-100 nm
Ingredient	SiO2
Product Code	NCZ-NP-620/24

Table S6: Relative enzyme activity

	**Activity (µM/min/mg protein)**	**Relative activity (%)**
Nano Chitosan Immobilized ASNase	454.55	186.37
Nano Gold Immobilized ASNase	384.62	157.70
Magnetic Iron(III) Oxide Nanoparticle Immobilized ASNase	416.67	170.84
Nano Silver Immobilized ASNase	370.37	151.85
Silica nanoparticles	357.14	146.43
Free Asparaginase	243.90	100

Relative activity (%) = Activity of immobilized enzyme /Activity of non-immobilized enzyme x 100

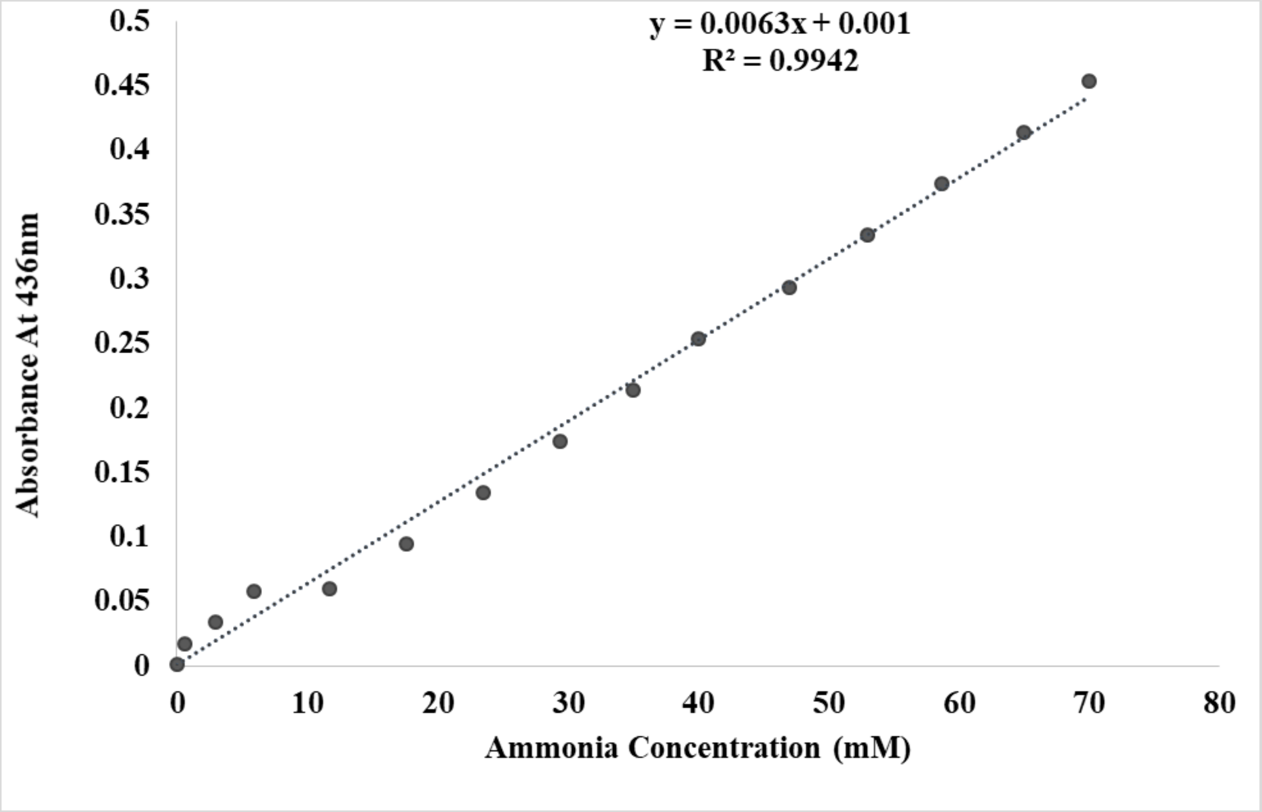

Figure S1: Ammonia calibration curve using Nesseler`s reaction
